# How private is private information? The ability to spot deception in an economic game

**DOI:** 10.1007/s10683-015-9474-8

**Published:** 2016-02-22

**Authors:** Michèle Belot, Jeroen van de Ven

**Affiliations:** 1 School of Economics, University of Edinburgh, Edinburgh, UK; 2 Tinbergen Institute, Amsterdam School of Economics, University of Amsterdam, Amsterdam, Netherlands

**Keywords:** Deception, Lie detection, Asymmetric information, Face-to-face interaction, Experiment, C91, D82, K4

## Abstract

We provide experimental evidence on the ability to detect deceit in a buyer–seller game with asymmetric information. Sellers have private information about the value of a good and sometimes have incentives to mislead buyers. We examine if buyers can spot deception in face-to-face encounters. We vary whether buyers can interrogate the seller and the contextual richness. The buyers’ prediction accuracy is above chance, and is substantial for confident buyers. There is no evidence that the option to interrogate is important and only weak support that contextual richness matters. These results show that the information asymmetry is partly eliminated by people’s ability to spot deception.

**Electronic supplementary material:**

The online version of this article (doi:10.1007/s10683-015-9474-8) contains supplementary material, which is available to authorized users.

## Introduction

The existence and consequences of asymmetric information are very prominent in economic analysis. Limitations to the transmission of information between a buyer and a seller often causes inefficiencies. Two assumptions that are typically made in the literature are responsible for this. First, people with private information will not disclose their information unless it is in their narrow self-interest to do so. This implies that they are prepared to lie.^1^ The second assumption is that the less informed people are not able to infer any private information beyond what they are able to infer from any equilibrium properties. If sellers always try to persuade buyers to purchase the most expensive item, or defendants always plead innocent, the less informed party cannot discriminate between honest or dishonest statements. In reality, though, there can be signals that (imperfectly) separate honest from dishonest people, and it is plausible that in some contexts people are able to process such signals and identify attempts of deception.

Despite its relevance for a wide range of economic situations, there is hardly any evidence in economics on the existence or relevance of signals that help people to detect deceit. In an intriguing experiment, Wang et al. (2010), show that in a sender-receiver game with private information, the senders’ pupils dilate more when their deception is larger in magnitude. In fact, they find that if the receiver could use the information on pupil dilation to predict the sender’s information, she could increase her payoff by 16–21 %. There is further evidence that other verbal and non-verbal cues may be systematically associated with deception (Ekman et al. 1988; Ten Brinke et al. 2012; Ekman 1988). A key question is to what extent people are able to *detect* these signals and spot deception. Recent work provides encouraging evidence in that respect (e.g., Belot et al. 2012), but only very little is known about the scope and conditions under which people can detect deceit.

The contribution of this paper is to examine how people’s ability to spot deception depends on the possibility to communicate and the contextual richness of the situation. Many studies show that the option to communicate has a large impact on behavior (see, e.g., Sally 1995). People frequently use communication to persuade others (Coffman and Niehaus 2015) and often become more trusting and cooperative when they can communicate. It is therefore interesting to study if communication helps them to assess the trustworthiness of others. For several reasons, communication is likely to affect the ability to detect deceit (Buller and Burgoon 1996). Questions can reveal inconsistencies in someone’s narrate, and elicit different behavioral responses by potential cheaters. These different responses can be verbal as well as nonverbal. The cognitive load to fabricate a consistent story is presumably higher when there is interaction, possibly resulting in increased leakages of signs of deception (DePaulo et al. 2003; Zuckerman et al. 1981). Similarly, the ability to detect deceit may depend on the richness of the context. With a richer context, there are more details or dimensions to talk about. Communicating about the outcome of a die roll is not the same as communicating about the possible defects of a car. A richer context provides people with more opportunities to mask any lies, but also to capture inconsistencies in someone else’s claims, since a richer context is by definition more complex. While both factors (communication and contextual richness) are plausible determinants of the ability to spot deception, the foregoing also shows that it is not clear if they will improve detection of deceit or make it worse.

In this study, we use data from a laboratory experiment to estimate people’s ability to detect lies in face-to-face encounters with free format communication.^2^ Face-to-face interaction remains one of the most popular tools of communication. For example, in a global survey among 2300 Harvard Business Review subscribers,^3^ respondents said that face-to-face meetings are key to building long-term relationships (95 %), negotiating contracts (89 %), meeting new clients (79 %), and understanding and listening to important customers (69 %). Relatedly, Eckel and Petrie (2011) show that in a laboratory setting people are willing to pay to be able to observe photos of their counterparts.

Our setting resembles an economically meaningful relationship. In the game that we implement, two participants are matched, one in the role of seller and the other in the role of buyer. The buyer can purchase either one of two products, but only the seller is privately informed about which of the two products matches the buyer’s interests. One of those products yields the seller a higher profit, and it is common knowledge which product that is. The experiment is set up such that the interests of the seller and buyer are sometimes aligned, so that the most profitable product matches the preferences of the buyer, while at other times there is a conflict of interest. Sellers are given the opportunity to make a recommendation and convince the buyer that the most profitable product matches the buyer’s interest. The buyer’s objective is to assess whether or not the seller is honest. The random variation in the alignment of interests allows us to infer the ability of buyers to detect deceit.

To study the effects of communication and contextual richness, we introduce two treatment variations. Our first treatment variation concerns the opportunity for the buyer to interrogate the seller. We implement a treatment in which sellers make a recommendation to buyers and buyers have an opportunity to interrogate the seller for a short period of time (Treatment “Questions”), and compare this to a treatment where buyers are not given this opportunity (Treatment “No-Questions”). Our second treatment variation alters the contextual details of the product. In the “Abstract” treatment, the product is simply a card that is either red or black. In the “Rich” treatment, the product is a holiday package. Sellers are provided with brief descriptions and pictures of two holiday packages, one labeled red and the other labeled black. Apart from providing context, the structure of the game is identical to the Abstract treatment.

Our findings provide support for an ability to detect deceit. Buyers are more likely to follow the seller’s recommendation when it is in their interest to do so. When we consider all sellers that recommend the product that is most profitable for them, the achieved accuracy is modest, although the impact on relative earnings is substantial. Averaged over all treatments, buyers are roughly 12 % points more likely to follow the seller’s recommendation when this is beneficial for them. In other words, sellers who have a good product to offer are 12 % points more likely to persuade the buyer that their product is good compared to sellers with a bad product. This translates into 24 % higher expected earnings for sellers who try to sell a valuable product compared to sellers who try to sell a bad product. If buyers were just randomly guessing, the expected difference in earnings would be zero. The achieved accuracy is more substantial for confident buyers. When buyers report a confidence in the correctness of their choice in the highest quartile, they are 19 % points more likely to follow the seller’s recommendation when this is beneficial for them. This translates into 37 % higher earnings for sellers that try to sell a valuable product compared to sellers that try to sell a bad product.

We do not find much support for the idea that the option to interrogate sellers increases the buyers’ accuracy in detecting deceit. We find weak support for an effect of contextual richness, but it is not robust across specifications. It is possible that our sample size is too small to detect this in a reliable manner.

The rest of the paper is organized as follows. In the next section we discuss the related literature. We describe the experimental design in Sect. 3. In Sect. 4, we present the results. Section 5 concludes.

## Related literature

Psychologists have intensively studied the ability of people to detect lies. From an evolutionary point of view it is not clear what we should expect about people’s abilities to detect deceit. The ability to deceive is evolutionarily advantageous (Dawkins and Krebs 1978; Wright 1995). And indeed, deception is widespread in nature. At the same time, natural selection will favor individuals that have the ability to accurately spot attempts of deceit (Dawkins 1978; Trivers 1985). Strategies to deceive others and to spot deception by others are ever evolving into more subtle and effective ways, and the human brain may even have evolved accordingly (Cosmides and Tooby 1992).

Empirical research shows that there are reliable verbal and nonverbal cues of deception, such as pupil dilation (Wang et al. 2010), type of smile (Ekman et al. 1988; ten Brinke et al. 2012), and high-pitch voice (Ekman 1988). Yet the consensus in the psychology literature is that untrained people without special equipment are poor lie detectors. The accuracy of deception rarely exceeds chance levels by an impressive margin, and only a small minority of studies finds an accuracy of at least 10 % points above chance (Bond and Depaulo 2008; DePaulo et al. 1985; Ekman and O’Sullivan 1991; Vrij 2008).

Several factors may have contributed to the findings in psychology. A typical drawback is that they do not allow for any interaction between potential deceivers and those who try to spot deception, or the interaction is at least partially based on predetermined transcripts (Hartwig et al. 2004). We conjecture that this may impede people’s ability to detect deceit. Most of these studies also do not provide incentives for observers to accurately spot deception, nor for the potential deceivers to successfully mislead observers, although there are some exceptions such as the studies by Frank and Ekman (1997) and Kraut and Poe (1980). Another limitation of these studies is that people are commonly instructed to tell the truth or a lie. This may create a bias in the accuracy of lie detection, as poor liars that would not normally attempt to deceive anyone are now asked to deceive, and people may feel less morally burdened if they are instructed to lie (see Belot et al. 2012, for a discussion of these and other limitations). Finally, many of these studies are focused on settings that have little to do with economically relevant situations.

There are only a few studies in economics that address the ability of people to detect deceit in face-to-face interactions. In some studies participants are allowed to communicate in a prisoner’s dilemma, and participants or observers are asked to make predictions regarding the behavior of others (Brosig 2002; Dawes et al. 1977; Frank et al. 1993). They find evidence that predictions are somewhat above chance levels. Ockenfels and Selten (2000) asked subjects to predict if participants in a bargaining experiment had low or high bargaining costs, where costs were randomly assigned to participants. The accuracy of predictions was above chance levels, which could be explained by objective features such as the length of the negotiations.

The most related study to ours is by Belot et al. (2012), who studied the ability to detect deceit in the context of a television game show where contestants play a high-stakes simultaneous prisoner’s dilemma game. Contestants take their decision after a round of free format communication. They asked independent subjects to predict the decisions (cooperate or defect) of the contestants before and after they communicate. They find evidence that the accuracy of predictions by observers are above chance levels. Observers rely on informative cues such as the contestant’s gender and promises. Specifically, they find that observers cannot distinguish true promises from lies when the promise was volunteered by the contestant, but they can, to some extent, when the promise was made after the game show host asked the contestant if (s)he would cooperate. These findings suggest that the format of communication—and in particular the ability to ask questions—may play a key role in the ability to detect deceit. Also closely related is the study by Coffman and Niehaus (2015). In their experiment, sellers can try to persuade buyers into buying certain products. They vary the communication possibilities for sellers, and show that communication helps sellers to increase the buyer’s valuations. They do not have an objective measure of the buyers’ value for products. Consequently, they cannot determine if sellers are misleading buyers or providing relevant information to them.

A few other studies analyze the accuracy of predictions when participants can send free-form written messages to each other. Chen and Houser (2013) analyze messages in what they call the “mistress game.” Interestingly, they find that messages contain some reliable cues of trustworthiness, such as the number of words and the mentioning of money, and they find that participants use those cues but not always in the correct manner. Utikal (2013) finds that participants can to some extent determine from a written apology whether or not an unfavorable action by the other person was intentional or accidental. These studies do not allow face-to-face interaction (thereby excluding the role of nonverbal signals) nor do they manipulate the communication possibilities.

Finally, there is also a line of research that examines if communication *per se* affects behavior. Most of the experiments with face-to-face communication study behavior in a prisoner’s dilemma (see Sally 1995). Written communication is studied in a variety of other games, including coordination games, cheap talk games, and hold-up problems (e.g., Charness and Dufwenberg 2011; Cooper et al. 1992; Ellingsen and Johannesson 2004a, b, amongst others). In those experiments, interactions are anonymous and the message space is sometimes (but not always) restricted to a limited number of possible messages. The objective of those studies is to examine if certain types of messages affect aggregate behavior, rather than examining if people can discriminate between the sincerity or dishonesty of a particular person based on cues in that person’s message or behavior.

To sum up, the main novelties of our study are (i) to study the ability to spot deception in a situation with face-to-face interaction, and to (ii) introduce exogenous variations in the length and format of the interaction (varying the ability to ask questions and the richness of the context of the transaction).

## Experimental design and procedures

### The game

Participants are matched into pairs of buyers and sellers. Sellers randomly draw a card from a deck that contained 5 red cards and 5 black cards. This draw determines if the red product or the black product is in the buyer’s interest to purchase. The color of the card remains private information to the seller and is not revealed to the buyer. In all treatments, there is a stage of 10 s face-to-face interaction during which sellers make a recommendation to the buyer to “purchase” the red product or the black product. The product (i.e., the card) never physically changes hands, but instead buyers are asked to write down in private if they want to purchase the red or the black product.

The payoff structure for the buyer and seller is common knowledge (see Table 1). The seller earns €20 if the buyer opts for the red product, independent of the card of the seller. The buyer earns €20 if she opts for the product that matches the color of the seller’s card. Thus, in terms of monetary payoffs, sellers are always better off if they can convince the buyers that they drew a red card, while the buyers are better off guessing the actual color of the seller’s card. The structure of this game reflects an important class of situations, in which it is common knowledge that sellers make higher profits by selling a particular brand, but they are also the only ones to know which product is truly in the best interest of the buyers.

**Table 1:** Payoff matrix

		Buyer’s choice	
		Red	Black
Seller’s card (random draw)	Red	20,20	0,0
	Black	20,0	0,20

This game has multiple equilibria. In all of the equilibria, the sender’s recommendation (“message”) is uninformative about the sender’s card. In Appendix 2, we show that under mild assumptions, the equilibrium outcome is unique. Specifically, if there is some positive fraction of credulous buyers who always take the seller’s recommendation at face value, or some positive fraction of sellers that are always honest, the unique equilibrium outcome is where all sellers claim that the card is red. However, as we shall see, our analysis of the buyer’s ability to spot deception does not depend on whether or not the senders’ claims are in accordance with equilibrium behaviour.

### Treatments

The above description is common to all treatments. In the “**No-Questions**” treatments, the buyer is not allowed to ask any questions. Sellers are given 10 s to make a recommendation. In the “**Questions**” treatments, we add another 90 s for the buyer to interrogate the seller. The communication in these 90 s is free format. Of course, to allow for any interrogation to take place, it was necessary to increase the length of the interaction. The possibility to ask questions and the length of the interaction therefore co-vary, and this should be kept in mind when we discuss the treatment effects. Alternatively we could have varied only one factor by imposing the same interaction time in the treatments without questions, but we felt that this would result in awkward and somewhat uneasy situations for participants.

In the “**Abstract**” treatment, the product description is simply a card that is either red or black. In the “**Rich**” treatment, the product is a holiday package. Sellers were provided with brief descriptions and pictures of two holiday packages, one labeled red and the other labeled black. One of these holiday packages is clearly better than the other (see the instructions in Appendix in online for an example) and in the best interest of the buyer. The color of the card drawn by the seller determines the color corresponding to this most attractive holiday package.

Apart from providing context, the structure of the game is identical to the Abstract treatment. Sellers are always better off selling the red holiday package, while the buyer’s best interest is to buy the most attractive package whose color is determined by a random draw that is private information to the seller. We provided each seller with a different set of holiday descriptions.

As mentioned in the introduction, it is not clear whether a richer context facilitates or hampers deceit detection. Sellers could use the context to mask their lies, but a richer context could also more easily reveal inconsistencies as it is more complex. In this setup, it is also possible that sellers have an easier time lying in the “Rich” treatment because they can focus on common characteristics between the two holiday packages.^4^

We used a 2 × 2 design giving us four different treatments. We label the four treatments as **NQ**-**A** (for **N**o-**Q**uestions, **A**bstract), **NQ**-**R**, **Q**-**A**, and **Q**-**R**. Every participant participated in only one of the context conditions (Abstract or Rich), but they all played in both interaction conditions (Questions and No-Questions). The order of the interaction condition was reversed between sessions.

### Procedures

Each session consisted of 20 rounds: 10 rounds in the No-Questions treatment and 10 rounds in the Questions treatment. In every round, a seller was re-matched to a new buyer. Sellers kept the same card for 10 rounds. Participants did not receive feedback until the end of the experiment. The setup and procedures were made common knowledge to the participants, except that we did not announce the treatment variation within a session on beforehand, but only announced that there would be a second part.

For each round we collected information about the recommendation made by the seller, the purchasing decision of the buyer, the confidence by sellers that the buyer would follow their recommendation, and the confidence by buyers that the seller drew a red card. The confidence statements were not incentivized. At the end of the experiment we collected some background information as part of a survey.

The main experiment took place at the Centre for Research in Experimental Economics and Political Decision Making (CREED) laboratory in Amsterdam between January and March 2012, October 2012, and February 2014, with a total of 310 participants divided over 16 sessions (47 % female, mean age 22).^5^ In five of the sessions we had 18 participants due to lower show up rates. All of the other sessions had 20 participants. Participants were students recruited from the CREED database. The CREED lab is an open space with cubicles.

Upon entering the room, participants were randomly assigned to their role as seller or buyer and they kept their role throughout the entire experiment. The instructions were distributed and read aloud by the experimenter. All participants received the same set of instructions (see Appendix in online). Participants were told that the experiment consisted of two different parts, and only received instructions for the second part after the first part had ended. Before the start of each part, sellers blindly drew a card from a deck with five red and five black cards. After showing the card to the experimenter, the experimenter would put the card back in the deck, shuffle the deck, and proceed to the next seller. The experimenter made note of the color that was drawn. The instructions explaining the game were framed in terms of a market, using terminology such as buying and selling. The descriptions of holiday packages were taken from a website (thomascook.com) and then slightly modified (an example is provided in the instructions).

The experiment was run using pencil and paper. The beginning and end of the rounds were announced by the sound of a bell. During the interactions, participants were asked to stand up. After every round, all sellers remain seated, and all buyers rotated in such a way that every buyer met every seller exactly once in each part. All participants were asked to keep their decisions sheets private. At the end of the experiment, one round was randomly selected for payment. We did not reveal which round was selected to ensure that participants could not identify the decision of any other participant, and participants were informed about this beforehand. Everybody received their earnings privately in an envelope. At the end of the experiment we also administered a short questionnaire, after all decisions had been made.

The experiment lasted for about 75 min. Average earnings were €18.80 including a fixed show up fee of €5.

## Results

### Recommendations

**Fig. 1:**
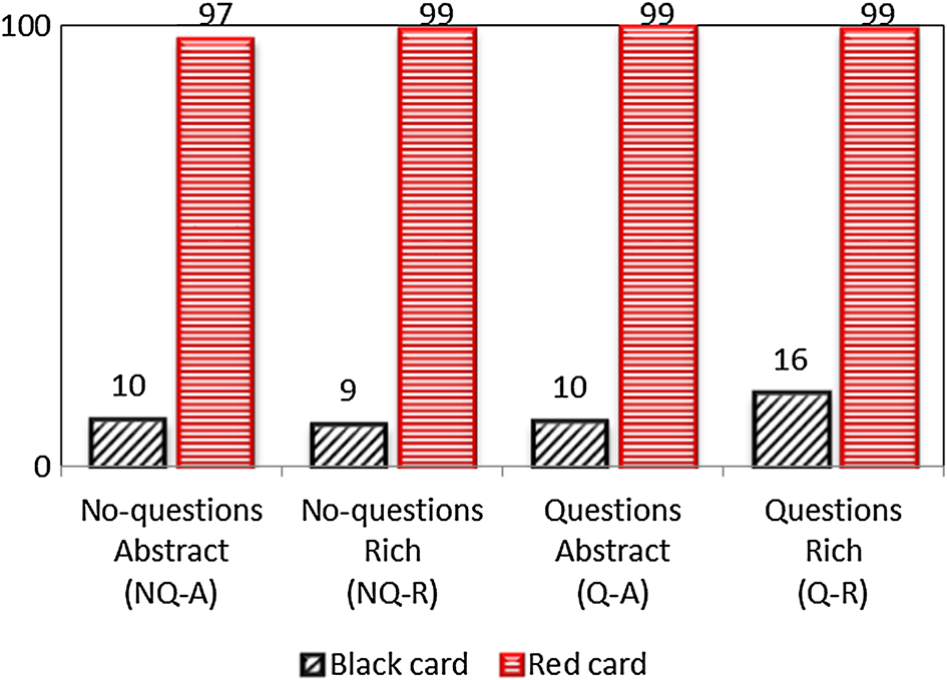
Percentage of truthful recommendations by treatment and the *color* of the seller’s card. (Color figure online)

We did not instruct sellers to lie or tell the truth, but with the stark incentives provided, we find that most sellers recommend the red product, whether it is in the interest of the buyer or not. Figure 1 shows the percentage of times that sellers make a truthful recommendation. Sellers with a red card recommend the red product almost 100 % of the time. Sellers with a black card recommend the black product about 11 % of the time.^6^ The percentage of black recommendations is somewhat higher in the treatment with questions and a rich context (Q-R) compared to the other treatments, although the difference is small and we do not find a significant difference across treatments (Kruskal–Wallis test, *p* = 0.454, adjusted for ties, taking the participants’ mean recommendation over all rounds within a treatment as the independent unit of observation). Nevertheless, the somewhat higher reluctance to lie in treatment Q-R can potentially result in a selection effect if only good liars make an attempt to deceive.^7^ We will discuss the role of selection later on.

**Fig. 2:**
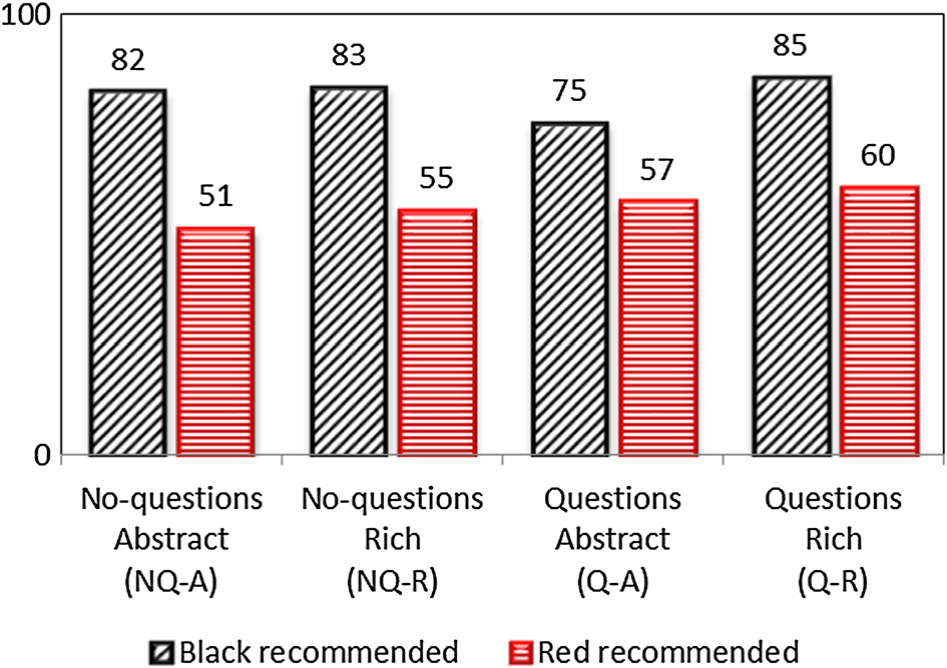
Percentage of buyers following the recommendation by treatment and recommended *color*. (Color figure online)

Do buyers follow the sellers’ recommendations? Figure 2 shows the percentage of buyers following the seller’s recommendation by treatment and recommended color. Averaged over all treatments, 81 % of buyers follow the seller’s recommendation if the recommendation is black, against 56 % if the seller recommends red. Buyers understand that a recommendation to buy black is very likely to be truthful, and are significantly more likely to follow this recommendation than the recommendation to buy red in all treatments (Wilcoxon rank sum test, two-tailed, *Z* = 4.457, *p* < 0.001 for treatment NQ-A; *Z* = 3.353, *p* < 0.001 for NQ-R; *Z* = 2.367, *p* = 0.018 for Q-A; *Z* = 3.691, *p* < 0.001 for Q-R).

It is not too surprising that buyers follow the red recommendation more than 50 % of the time. If they are in doubt, they may simply purchase the red product out of efficiency concerns. Furthermore, given that some senders with a black card are honest, sellers with a red card are overrepresented among sellers that recommend red. Thus, following the red recommendation gives buyers a slight edge in getting it right. More remarkable is that not all buyers follow the black recommendation. These buyers may think that sellers are trying to mislead them by telling the opposite of what they aim for, expecting that their recommendation will not be followed. Such a strategy is often used in other games (see, e.g., Sutter 2009), even though there is little rationale for it in the current setting. Buyers indeed report not to be convinced by the truthfulness of a black recommendation. Their average confidence that the card is black is high, even if not maximal (7.3, on a scale from 0 to 10). Buyers who purchase red after a black recommendation appear suspicious, however. Their average reported confidence that the card is black is 4.4, i.e., they believe that the card is more likely to be red than black. Alternatively, buyers may understand that a black recommendation is likely to be truthful, and reciprocate the seller’s honesty by purchasing red. We believe that this latter explanation is somewhat unlikely given the steep incentives for making the correct decision. In any case, although the percentage of buyers not following a black recommendation is substantial (19 %), the mistake is on average not very costly given the low frequency with which sellers recommend black.

### Detecting deceit

Our main question is whether or not the accuracy of buyers’ predictions is above chance levels. Our strategy to determine if buyers can spot deception is to compare the proportion of times that a buyer buys the red product when the seller has a red card, P(R|R), to the proportion of times that a buyer buys the red product when the seller has a black card, P(R|B). Our measure of the ability to detect deceit is thus given by:

1 $$ D = P\left( {R |R} \right) - P\left( {R |B} \right). $$

If a buyer cannot identify signals of deceit, then the proportion of times that she buys the red product is independent of the card drawn by the seller, and consequently *D* will be 0. By contrast, *D* is positive if sellers are leaking signals of deception, and equals 1 if buyers can perfectly discriminate honest from deceitful sellers. A negative value of *D* indicates a worse than chance accuracy.^8^ The size of *D* also has a direct interpretation: it reflects the difference in proportions of selling a red card between sellers with a red card and sellers with a black card. For instance, if *D* = 0.1, sellers with a red card can expect to sell 10 % points more red cards than a seller with a black card. Or, equivalently, buyers would be 10 % points more likely to buy the red card when this is in their best interest compared to when it is not in their best interest.

Note that our measure *D* is insensitive to the proportion of sellers with a red card and the proportion of times that a buyer buys the red product, in the sense that random guessing by buyers results in *D* = 0 while any positive or negative *D* indicates non-random guessing. Thus, some buyers may be more inclined to buy red than black if they care about the seller’s payoff, but this will not lead to a positive value of *D* if they are guessing randomly. There might be a downward bias in *D*, however, if there are buyers who can guess better than chance but who decide to buy the red product whenever they are in doubt. If anything, we may therefore underestimate buyers’ ability to detect deceit.

We find evidence that buyers can detect deceit, though the effect size tends to be modest. Table 2 shows the proportion of buyers that buys the red card, depending on whether the seller has a black or a red card. We also report the *p* values for the test that *D* = 0 (Wilcoxon signed rank tests over the mean choices of participants,^9^ two tailed). We first consider the entire sample of sellers (Panel A of Table 2). In all treatments, buyers are significantly more likely to buy the red card when this is in their best interest: the measure *D* is positive and significantly different from zero in all treatments. The accuracy of detection ranges from 11 % points (*D* = 0.11) to 18 % points (*D* = 0.18). The accuracy appears to be higher in the treatments with a rich context. If we pool treatments with a rich context, we find that the accuracy is marginally significantly higher in those treatments than in treatments with an abstract context (Mann–Whitney test, *Z* = 1.735, *p* = 0.083).

**Table 2:** Proportion of buyers buying red (by treatment)

Treatment	No questions		Questions	
	Abstract	Rich	Abstract	Rich
Panel A: all sellers				
Seller has black card, P(R|B)	0.44	0.44	0.50	0.46
	(0.03)	(0.03)	(0.03)	(0.03)
Seller has red card, P(R|R)	0.55	0.62	0.62	0.64
	(0.03)	(0.03)	(0.03)	(0.03)
Accuracy of detecting deceit (*D*)	**0.11**	**0.18**	**0.12**	**0.18**
Test *D* = 0 (*p* value)	0.003	0.001	0.005	0.001
Panel B: sellers recommending red				
Seller has black card, P(R|B)	0.47	0.49	0.53	0.51
	(0.03)	(0.04)	(0.03)	(0.05)
Seller has red card, P(R|R)	0.56	0.63	0.62	0.65
	(0.03)	(0.03)	(0.03)	(0.04)
Accuracy of detecting deceit (*D*)	**0.10**	**0.14**	**0.09**	**0.14**
Test *D* = 0 (*p* value)	0.013	0.001	0.048	0.005

Accuracy of detecting deceit measured as $D = P\left( {R|R} \right) - P\left( {R|B} \right)$. Standard errors in parentheses. *p* values of test *D* = 0 based on two-tailed Wilcoxon signed-rank tests with the mean of a participant over all rounds as the independent unit of observation

It is relatively easy for buyers to judge the honesty of sellers when they recommend the black product. As a more stringent test, we exclude all cases in which sellers recommended black in Panel B of Table 2. This lowers the accuracy of detection somewhat, now ranging from 9 to 14 % points depending on the treatment, but the accuracy remains significantly different from zero in all treatments. The accuracy still tends to be slightly higher with a rich context, but the difference is not significant (Mann–Whitney test, *Z* = 1.136, *p* = 0.256).^10^ Since we found that sellers with a black card were somewhat more reluctant to lie in treatment Q-R, any comparison between this treatment and the other treatments could in principle be partly due to a selection effect, if only the good liars attempt to deceive in that treatment. Given that the difference in lying rates between treatments is small, and given that the ability to detect deceit is comparable to that in the other treatments, we have no indication that such selection effects are important.^11^

Overall, we find evidence that buyers can detect deceit. We do not find support for the idea that interaction is of importance for the ability to detecting deceit, and only weak support for the idea that contextual richness is relevant. The absence of a significant treatment effect is, of course, no proof that there is no treatment effect. Possibly, our sample size is too small to reliably detect a treatment effect.

Although the effect size tends to be modest, it can still be economically relevant. Among the entire sample of sellers, buyers are 15 % more likely to purchase red if the seller has a red card compared to sellers with a black card (61 versus 46 %). This is a 32 % increase in expected earnings for sellers with a red card compared to sellers with a black card. Even when we restrict the sample to sellers that recommend red, sellers with a red card are 12 % points more likely to convince buyers to choose red compared to sellers with a black card (62 versus 50 %), a 24 % increase in expected earnings for sellers.

A regression analysis based on individual choice data by and large confirms these results. Table 3 reports regressions where the dependent variable is the buyers’ decision to choose the red product. The focus is again on cases in which sellers recommended red, to provide a more stringent test (Table 5 in Appendix 1 reports the estimates for the full sample of sellers). The independent variables of interest are the interactions of the treatment and whether the seller has a red card (Treatment × red card seller). We also included treatment dummies as controls. We estimate a linear model with buyer and seller random effects.^12^ If a coefficient of the interaction term is positive, it shows that in those treatments a buyer is more likely to buy the red product if the seller has a red card compared to when the seller has a black card. This is the same as measure *D*. A positive coefficient therefore signals that buyers can detect deceit in a particular treatment.

**Table 3:** Buyer buying red when sellers recommend red

Dependent variable: buyer buys red	(1)	(2)	(4)	(5)
Sample	All rounds	All rounds	Rounds 6–10	Confident buyers
Seller has red card (1)	0.085** (0.037)	0.097** (0.041)	0.065 (0.055)	0.194** (0.081)
Treatments questions × seller has red card (2)	−0.006 (0.041)	−0.030 (0.057)	−0.028 (0.077)	−0.035 (0.106)
Treatments rich × seller has red card (3)	0.061 (0.042)	0.036 (0.059)	0.117 (0.079)	0.091 (0.110)
Treatment questions & rich × seller has red card (4)		0.051 (0.082)	−0.011 (0.111)	−0.056 (0.145)
Test treatment × red card = 0 (*p* values)				
NQ-R: (1) + (3) = 0		0.002	0.001	0.000
Q-A: (1) + (2) = 0		0.108	0.497	0.023
Q-R: (1) + (2) + (3) + (4) = 0		0.000	0.011	0.004
Controls	Yes	Yes	Yes	Yes
Observations	2785	2785	1363	682
Number of buyers	155	155	155	122

Dependent variable: buyer buys red. Two-way linear random effects model (allowing for buyer and seller random effects). Sample is sellers recommending the red product. Confident buyers are cases in which the confidence of the buyer is in the top quartile. Control variables: Treatment dummies. Standard errors in parentheses

*** *p* < 0.01, ** *p* < 0.05, * *p* < 0.1

In specification (1) we include a dummy variable indicating whether or not the seller has a red card, and dummy variables to examine the effects of the treatments (Questions and Rich context). Buyers are 8.5 % points more likely to purchase the red card when the seller has a red card. The effect is smaller in the questions treatments, but the coefficient is very small. The effect is 6 % points larger for the rich context treatments, though this effect is not significant (the *p* value is 0.151).^13^ In specification (2) we include an interaction between Questions and Rich, but the interaction term is not significant. This specification also allows determining the accuracy for each of the treatments separately. We report the *p* values associated with the tests that the achieved accuracy is zero (Treatment × red card = 0). For instance, to test if the accuracy is positive in treatment NQ-R, we test whether we can reject that the sum of coefficients (1) and (3) is equal to zero. Coefficient (1) by itself shows the accuracy in treatment NQ-A. Accuracy is significantly above chance in all treatments except for treatment Q-A, where it is marginally insignificant with a *p* value of 0.108.

### Learning

A natural question is whether any learning takes place. The scope for learning is somewhat restricted in our experiment, because participants do not receive feedback on their decisions. In the treatments without interrogation we do not expect to see learning for that reason. Conceivably some learning can take place in the Question treatments: with experience, sellers may become more convincing in their recommendations, and buyers may learn to ask more relevant questions. Thus, a priori, the ability to detect deceit could increase or decrease over time.

**Fig. 3:**
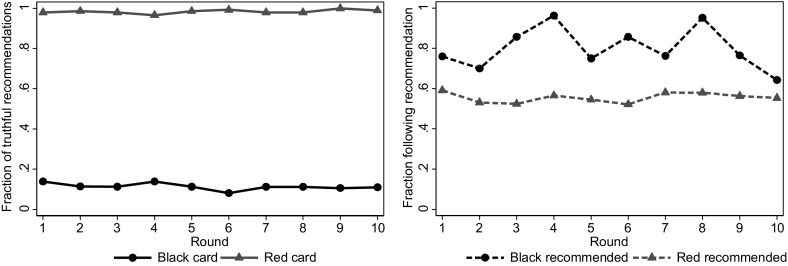
Recommendations and decisions over time. *Left panel* fraction of cases in which sellers make a truthful recommendation by the *color* of the card. *Right panel* fraction of cases in which buyers follow the seller’s recommendation by the *color* of the recommendation. (Color figure online)

We start by examining the development of sellers’ and buyers’ behavior over time, aggregating over all treatments. The patterns are rather stable over the rounds. The left panel of Fig. 3 shows the fraction of times that sellers make truthful recommendations. Sellers with a red card consistently recommend red, while those with a black card recommend black about 11 % of the time in every round. The right panel shows the fraction of times that buyers follow the seller’s recommendation. Buyers are more likely to follow a black recommendation than a red recommendation in each round. The fraction of buyers following a black recommendation naturally fluctuates a bit more over the rounds, as the number of observations is relatively small. There are no discernible time trends in behavior.

**Fig. 4:**
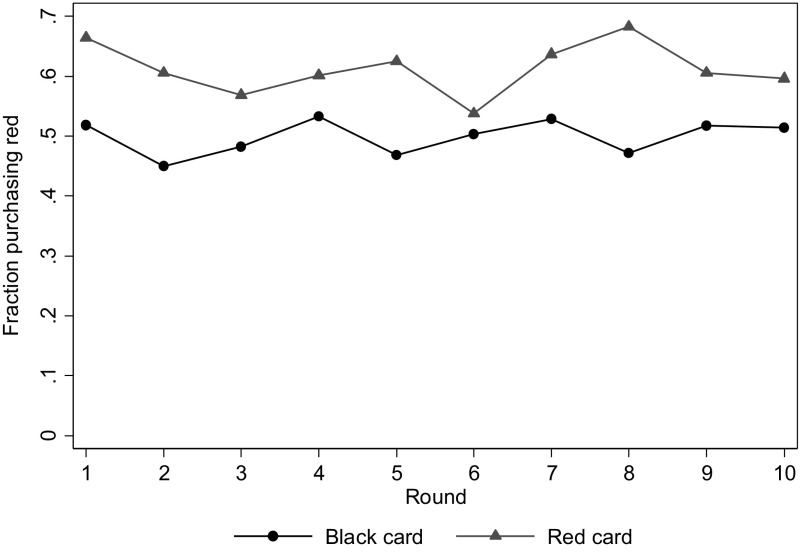
Accuracy of detecting deception over time. Fraction of buyers purchasing *red* by the color of the seller’s card. (Color figure online)

Figure 4 shows how the accuracy to spot deception develops over the rounds. Buyers are consistently more likely to purchase the red product if the seller has a red card compared to when the seller has a black card. The gap between the lines (i.e., the measure *D*) remains relatively constant over the rounds, revealing no time trend in the accuracy to spot deception. A similar conclusion can be derived from Column (3) of Table 3. This specification shows the estimates of the accuracy for the second half of each treatment. The results are similar to those of column (2), except that in treatment NQ-A we no longer find evidence of buyers detecting deceit. Another robustness check is to test for order effects. Table 6 in Appendix 1 reproduces Table 2 of the main text, only including participants in their first treatment. The significance levels are naturally lower with this smaller sample, but the effect sizes remain similar. The only exception is the higher accuracy in treatment NQ-R when sellers recommend red (*D* = 0.20, see Panel B of Table 6). On net, there seems to be little learning.^14^

### Confidence

Buyers vary in their degree of confidence when they meet different sellers. In our setup, the buyers’ confidence is not relevant because they are forced to buy one of the two products. It seems plausible, however, that in other contexts this may be relevant, because the buyer’s confidence may affect his or her decision to purchase to product or not. For instance, perhaps only buyers who are very confident in the truthfulness of the seller’s claims will proceed to purchase the product, while other buyers will refrain from making a purchase. It is therefore interesting to examine if the buyer’s confidence is justified. In column (4) of Table 3 we restrict the sample to encounters in which buyers report to be confident in the correctness of their choice. We categorize a choice as confident if the reported confidence is in the highest quartile. We find that the achieved accuracy is higher and substantial in all treatments, varying between 16 and 29 % points. It is not entirely clear what drives these results. It could simply be that some buyers are better than others at reading signals of deceit (even if senders leak the same signals towards all buyers), and are aware of their skill. Another possibility is that more confident buyers induce sellers to leak signals (e.g., by asking the right questions) that would not have leaked in the presence of other buyers.^15^ Our data does not allow us to disentangle between these two explanations.

**Fig. 5:**
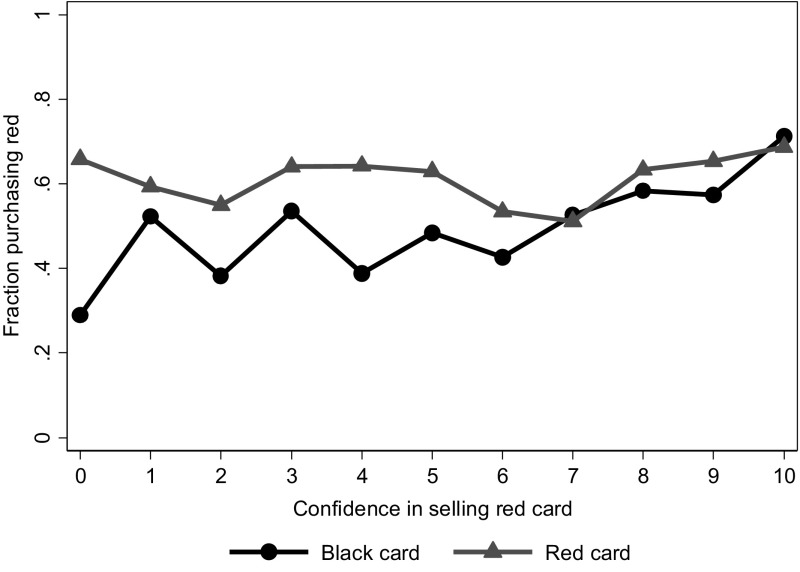
Fraction of buyers purchasing *red* by seller’s confidence and color of the card. On the horizontal axis is the seller’s confidence in the buyer purchasing *red*. Sample is sellers recommending *red*. (Color figure online)

Similarly, sellers vary in their confidence of having convinced the buyer to purchase the red product. Figure 5 shows how successful sellers are in selling the red product depending on their confidence level and the color of their card. For sellers with a red card, there is not much correlation between their confidence and the percentage of times that the buyer purchases the red product (Pearson’s rho 0.023, *p* = 0.381).^16^ By contrast, for sellers with a black card there is a positive correlation: confident sellers are indeed more likely to have convinced buyers to purchase the red product (Pearson’s rho 0.133, *p* < 0.001).

### Individual characteristics

Table 4 shows the results of a regression of a correct decision by a buyer (i.e., a buyer that buys the color matching that of the seller) on several individual characteristics of the participants. Individual characteristics are organized by the role of participants. If certain characteristics are positively (/negatively) related to the ability to detect deceit, then we should expect the coefficients of these variables to be positive (/negative) for buyers. If certain characteristics are positively (/negatively) related to the ability to deceive, we should expect the coefficients of these variables to be negative (/positive) for sellers.

**Table 4:** Accuracy of choices and individual characteristics

Dep. variable: correct prediction	(1)	(2)	(3)	(4)
Seller’s card	Black card	Red card	Black card	Red card
Buyer characteristics				
Female	−0.039 (0.047)	−0.011 (0.044)	−0.038 (0.054)	−0.010 (0.053)
Self-perceived ability to detect deceit	0.016 (0.016)	0.004 (0.015)	0.022 (0.018)	0.014 (0.018)
Frequency poker	−0.004 (0.027)	0.029 (0.027)	−0.001 (0.031)	0.043 (0.032)
Seller characteristics				
Female	0.019 (0.046)	0.008 (0.041)	0.022 (0.052)	0.049 (0.048)
Self-perceived ability to deceive	−0.009 (0.011)	−0.001 (0.010)	−0.011 (0.012)	0.001 (0.011)
Frequency poker	−0.043 0.019	−0.017 0.008	−0.042 (0.030)	0.009 (0.028)
Joint characteristics				
Both female	0.057 (0.053)	0.020 (0.051)	0.071 (0.060)	0.001 (0.062)
Shared language			0.028 (0.036)	0.071** (0.036)
Friends or acquaintance			−0.103 (0.066)	0.184*** (0.063)
Observations	1368	1404	1086	983
Number of buyers	154	154	116	116

Two-way linear random effects model (allowing for buyer and seller random effects). Sample is sellers recommending the red product

Standard errors in parentheses

*** *p* < 0.01, ** *p* < 0.05, * *p* < 0.1

The characteristics we include are gender and a dummy variable that equals 1 if the buyer–seller pair in a particular round consists of two females. We also include a self-reported measure of the participant’s perceived own ability to deceive or spot deception (both on a seven point Likert scale, ranging from 1: not good at all, to 7: very good) and how often they play poker (on a five scale ranging from 1: never, to 5: once or twice a day).

We report the results separately for sellers with a black card and sellers with a red card. Because we found no strong statistical difference in accuracy between treatments, we pool all treatments. Consistent with results from a meta-study (Bond and Depaulo 2008), for most variables we do not find a strong indication that they are related to the ability to deceive or spot deception. The coefficients are all insignificant and modest in size. We do, however, find a positive effect of “shared language” and “friendship or acquaintance” (see Columns 3 and 4). Somewhat surprisingly, this is only true when sellers have a red card. Thus, it is not the case that sellers with a black card are less convincing liars towards friends. Rather, they are more likely to convince buyers that they have a red card when they are honest about it. When we estimate the effects separately for each treatment (not reported), we find that the effects of shared language and friends are driven by one treatment only (NQ-R), so these results should be taken with caution.

Finally, there could be some unobserved characteristics that make some sellers or buyers particularly good or bad at deceiving or spotting deception. We first examine this by comparing participants’ performances between the two parts, again focusing on cases in which the seller recommended the red product. For buyers, we look at the number of correct guesses within each part. The correlation between the two parts is very low (0.018) and not significant (Spearman’s rho, *p* = 0.827). For sellers, we look at the number of red cards that they managed to sell within each part. The correlation is 0.235 and significantly different from zero (*p* = .004). Thus, the performance of sellers in part 1 is somewhat predictive of their performance in part 2.

**Fig. 6:**
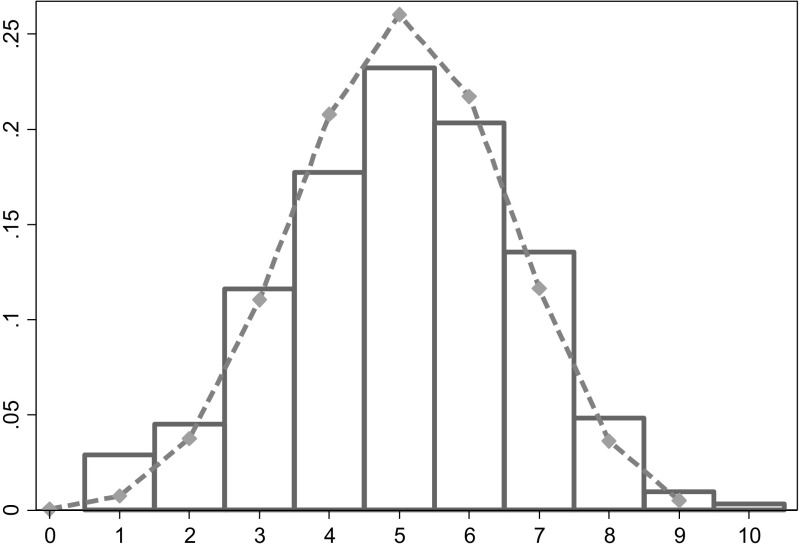
Histogram of number of correct guesses by buyers when sellers recommend red. The *bars* represent the actual distribution, the dashed line the expected distribution if all buyers are the same. (Color figure online)

Another way to examine this question is to look at the distributions of performance. Figure 6 shows the histogram of the number of correct guesses by buyers. Some buyers performed much better than other buyers. Of course, even if they all have the same ability to spot deception, chance will result in differences in performance. We therefore compare the actual distribution to the expected distribution that would result under the assumption that all buyers have the same ability to spot deception. Averaged over all treatments, buyers purchase red with probability 0.5 when they meet a seller with a black card and with probability 0.62 when they meet a seller with a red card. The dashed line shows the resulting expected distribution based on those probabilities.^17^ The actual and expected distributions are very close.

**Fig. 7:**
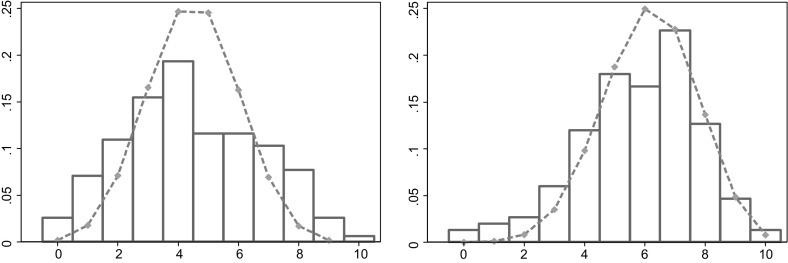
Histogram of number of *red* *cards* sold when sellers recommend *red*. The *bars* represent the actual distributions, the *dashed lines* the expected distribution if all sellers are the same. *Left* sellers with a *black* *card*. *Right* sellers with a *red* card. (Color figure online)

The picture looks a bit different for sellers. Figure 7 shows the actual and expected distribution for sellers with a black card (left) and sellers with a red card (right). For sellers with a red card, the expected distribution resembles the actual distribution. For sellers with a black card, however, the actual distribution has less mass in the middle compared to the expected distribution. There appear to be more sellers with a poor performance and more sellers with a good performance than could be expected to happen by chance if all sellers were the same.

## Discussion

Our results are relevant for a wide range of economic exchange situations. Economists have extensively studied markets with asymmetric information, in which some parties are better informed and have objectives that are not fully aligned with those of the other parties. Salesmen, financial advisors, and manufacturers of products are all better informed about the products they sell than their vulnerable customers. The predominant view is that this causes inefficiencies in, or even nonexistence of, markets (Akerlof 1970). Our results show that the information asymmetry is partly eliminated by people’s ability to spot deception, although the achieved accuracy of detecting deceit is nowhere near perfect; people still often buy lies. We find at best some weak support for the idea that contextual richness matters. The option to interrogate does not improve the accuracy. This is especially interesting given that face-to-face interactions are highly valued in business (Harvard Business Review 2009). One possibility is that many people are overconfident in their ability to spot deception, and therefore prefer face-to-face meetings even if it does not help them to spot deception. Of course, there are many other differences between our experimental setup and actual business meetings, and the ability to spot deception might be larger under some conditions.

The absence of strong treatment effects can be due to several factors. We already argued that the predicted effects were ambiguous. For instance, the contextual richness can help to mask lies or to expose inconsistencies. Possibly, those effects more or less offset each other. Alternatively, the payoff structure that we implemented might have played a role. Some sellers with a black card may have thought that lying was justified, since it was either the buyer or the seller that would receive a positive payoff. This may have taken away feelings of guilt in deceiving others, thereby making detection of deceit harder.

We close our paper by mentioning some limitations of our setup. Although we have established the importance of some factors in the ability to spot deception, we are uncertain about the cues that buyers use. Answering that question would require experiments where different communication channels are studied in isolation, and/or where conversations are recorded and categorized. The coding of conversations has proven valuable in different contexts, shedding light on the strategies used by participants (see, e.g., Brandts et al. 2012; Charness and Dufwenberg 2006; Cooper and Kuhn 2014; Coffman and Niehaus 2015). A possible concern is that such recording could affect the behavior of participants in a non-trivial way, though participants in Cooper and Kuhn (2014) show little hesitance to lie.

Another interesting question is whether buyers and sellers with certain characteristics will self-select on markets. Perhaps in real markets sellers are relatively effective at deceiving people, or perhaps people will only buy a product if they can trust the seller. Furthermore, in our experiment buyers are aware of the possibility of deception, whereas in real life they may be more credulous (Irlenbusch and Ter Meer 2013). Another aspect of our design is that the private information of sellers concerns the state of nature, rather than their own actions. Serra-Garcia et al. (2013) show that people are less likely to lie about their actions than about their knowledge of the state of nature. Clearly, many extensions are possible, and we hope that our framework provides an avenue for future research. Given the limited knowledge that is currently available, we believe that this is an important topic that deserves more study (Ottaviani and Squintani 2006).

### Electronic supplementary material

Below is the link to the electronic supplementary material.

Supplementary material 1 (DOCX 167 kb)

## Appendix 1: Robustness checks

See Tables 5 and 6.

**Table 5:** Buyer buying red for any recommendation

Dependent variable: buyer buys red	(1)	(2)	(3)	(4)
Sample	All rounds	All rounds	Rounds 6–10	Confident buyers
Seller has red card	0.092** (0.036)	0.103** (0.041)	0.082 (0.054)	0.301*** (0.098)
Treatments questions × seller has red card	0.021 (0.040)	−0.003 (0.056)	−0.016 (0.075)	0.055 (0.130)
Treatments rich × seller has red card	0.068 (0.042)	0.043 (0.058)	0.126 (0.077)	0.084 (0.133)
Treatment questions & rich × seller has red card		0.050 (0.081)	−0.014 (0.108)	−0.056 (0.174)
Test equality of coeffcients (*p* values)				
(1) + (3) = 0 (NQ-R)		0.000	0.000	0.000
(1) + (2) = 0 (Q-A)		0.015	0.228	0.000
(1) + (2) + (3) + (4) = 0 (Q-R)		0.000	0.001	0.000
Controls	Yes	Yes	Yes	Yes
Observations	2995	2995	1452	420
Number of buyers	155	155	155	112

Dependent variable: buyer buys red. Two-way linear random effects model (allowing for buyer and seller random effects). Sample is all sellers. Confident buyers are cases in which the confidence of the buyer is in the top quartile. Control variables: Treatment dummies

Standard errors in parentheses

*** *p* < 0.01, ** *p* < 0.05, * *p* < 0.1

**Table 6:** Accuracy of buyers’ decisions in part 1

Treatment:	No questions			Questions	
	Abstract	Rich		Abstract	Rich
Panel A: all sellers					
Accuracy of detecting deceit (*D*)	**0.09**	**0.20**		**0.11**	**0.20**
Test *D* = 0 (*p* value)	0.117	0.001		0.106	0.004
Panel B: sellers recommending red					
Accuracy of detecting deceit (*D*)	**0.10**	**0.20**		**0.08**	**0.16**
Test *D* = 0 (*p* value)	0.138	0.001		0.199	0.037

Accuracy of detecting deceit measured as $D = P\left( {R|R} \right) - P\left( {R|B} \right)$. Sample is participants in their first treatment

Standard errors in parentheses

*p* values of test *D* = 0 based on two-tailed Wilcoxon signed-rank tests with the mean of a participant over all rounds as the independent unit of observation

## Appendix 2: Equilibrium of the game

In this Appendix, we show that the game has a continuum of Nash equilibria, but that the equilibrium outcome is unique once we impose some mild restrictions, i.e., the existence of credulous buyers or honest sellers.

Suppose the true state of nature is red or black: $\omega \in \Omega =\{R,B\}$. Each state is equally likely to occur. The state is privately observed by sellers. Sellers make a claim about the state of nature: $m \in M=\{{\hat{R}},{\hat{B}}\}$. After that, buyers make a guess about the state of nature. A seller earns $\pi ^s>0$ if the buyer guesses red, and 0 otherwise. A buyer earns $\pi ^b>0$ if his or her guess matches the state of nature, and 0 otherwise. In our experimental setup, $\pi ^s=\pi ^b=20$. Let $q_m(\omega )$ be the probability that a seller claims *m* when the true state is $\omega$. Let $\lambda _{\omega }(m)$ be the probability that the buyer guesses $\omega$ after claim *m* by the seller.

This game has a continuum of Nash equilibria, all characterized by uninformative (“babbling”) claims by sellers.

### **Proposition 1**

*In every equilibrium,* $q = q_{\hat{R}}(R)= q_{\hat{R}}(B)$ *, and either (i)* $q \in (0,1)$ *and* $\lambda = \lambda _{R}(\hat{R}) = \lambda _{R}(\hat{B})$ *, or (ii)* $q=1$ *and* $\lambda _{R}(\hat{R}) \ge \lambda _{R}(\hat{B})$ *, or (iii)* $q=0$ *and* $\lambda _{R}(\hat{R}) \le \lambda _{R}(\hat{B})$.

### *Proof*

It is easy to see that *q* must be 1 if $\lambda _{R}(\hat{R}) > \lambda _{R}(\hat{B})$ and 0 if $\lambda _{R}(\hat{R}) < \lambda _{R}(\hat{B})$. If $\lambda _{R}(\hat{R}) = \lambda _{R}(\hat{B})$, sellers are willing to randomize. That $q = q_{\hat{R}}(R)= q_{\hat{R}}(B)$ follows from the fact that if $q_{\hat{R}}(R) \ne q_{\hat{R}}(B)$ buyers are not willing to randomize guesses with equal probabilities after each claim, in which case sellers have a dominant strategy. $\square$

We will show that the set of equilibria is greatly reduced if we assume that there exist credulous buyers. We will define a credulous buyer as a buyer that always takes the seller’s claim at face value and follows the recommendation (cf. Ottaviani and Squintani 2006).

We also make the following assumptions:

### **Assumption 1**

$q_{\hat{R}}(R) \ge q_{\hat{R}}(B)$: The likelihood that a seller claims that the state is red is at least as high in the red state as in the black state.

This assumption rules out perverse equilibria in which buyers should interpret the claim “red” as most likely coming from sellers that observed the state “black” and vice versa. It is akin to a natural language assumption and is consistent with the data. Similarly:

### **Assumption 2**

$\lambda _{R}(\hat{R}) \ge \lambda _{R}(\hat{B})$: The likelihood that a buyer buys red is at least as high after a red claim as after a black claim.

### **Proposition 2**

*Let* $\alpha$ *be the fraction of credulous buyers in the population. For any* $\alpha >0$ *, the unique equilibrium outcome satisfying Assumptions* 1 *and *2 *is where sellers always claim that the state is red*: $q_{\hat{R}}(B)=q_{\hat{R}}(R)=1$.

### *Proof*

Suppose first that $q_{\hat{R}}(R) > q_{\hat{R}}(B)$. Then all buyers will guess red when a seller claims red, and guess black when a seller claims black. Thus, sellers will deviate when the state is black. Suppose next that $q_{\hat{R}}(R) = q_{\hat{R}}(B)$. Claims by sellers are uninformative, and in that case, non-credulous buyers are indifferent between guessing black and red, and are willing to randomize. Sellers always like to claim red, because it gives an expected payoff of $\alpha \pi ^s +(1-\alpha )\lambda _{R}(\hat{R}) \pi ^s$, while claiming black only yields an expected payoff of $(1-\alpha ) \lambda _{R}(\hat{B}) \pi ^s$. $\square$

Similarly, the set of equilibria is greatly reduced when a fraction $\gamma$ of sellers is “unconditionally honest,” telling the truth no matter what response they expect from the buyers, while the other “opportunistic” sellers still choose the strategy that maximizes their expected payoffs.

### **Proposition 3**

*Let* $\gamma$ *be the fraction of unconditionally honest sellers in the population. For any* $\gamma >0$ *, the unique equilibrium satisfying Assumption 1 is where opportunistic sellers always claim that the state is red*.

### *Proof*

Consider a red claim. Then the buyer’s posterior that the state is red equals $[\gamma +(1-\gamma ) q_{\hat{R}}(R)]/[\gamma +(1-\gamma ) q_{\hat{R}}(R)+(1-\gamma ) q_{\hat{R}}(B)]$. For any $q_{\hat{R}}(R) \ge q_{\hat{R}}(B)$, the posterior is above $\frac{1}{2}$, and thus buyers will guess red. After a black claim, the posterior that the state is red is below $\frac{1}{2}$, and thus buyers will guess black. Thus, all opportunistic sellers wish to claim red. $\square$

The logic behind this result is that among all sellers claiming red, the proportion of opportunistic sellers who observed red is at least as high as those who observed black, and they mix in with the unconditionally honest sellers that observed (and claimed) red. Hence, the sellers who truly observed red are in the majority, and thus buyers will guess red after a red claim. Similarly, after a black claim, the sellers who truly observed black are in the majority and buyers will guess black.

## Footnotes

1 Several studies show that people are sometimes averse to lying (e.g., Gneezy 2005; Charness and Dufwenberg 2006) and analyze the consequences this may have (e.g., Kartik 2009; Kartik et al. 2007).

2 As a shorthand we will adopt the terminology of lie detection, but strictly speaking it would be more appropriate to speak about *information transmission* in our setting. Information transmission is a broader concept that includes the detection of lies.

3 Harvard Business Review (2009). “Managing Across Distance in Today’s Economic Climate: The Value of Face-to-Face Communication”.

4 We thank an anonymous referee for pointing this out.

5 We first ran 8 sessions in 2012. The sessions in 2014 were meant as a self-replication study. We could not replicate some of our earlier findings. We present the aggregate results from the two sets of sessions, and indicate where the results differ. Prior to this experiment, we also ran four sessions with 78 participants as part of a class in experimental economics (January 2012). Participation was compulsory for those students, and at a few instances buyers played in pairs. The incentives were the same, but they did not receive a show-up fee. Because this is a different subject pool (where people are more likely to know each other) we do not include them in the results reported here. Most of our results are robust to including those observations. We will indicate where the results differ in an important way.

6 Sellers that recommend black tend to use a mixed strategy. Only very few sellers consistently recommend the black card when they have a black card. There are 43 cases in which a seller recommends black in at least one of the 10 rounds. Of those, there are 6 cases in which a seller recommends black in all 10 rounds. In most other cases, sellers recommend black in only 1–3 rounds (24 out of 43 cases).

7 Konrad et al. (2014) study self-selection and find evidence that only good liars attempt to deceive when the consequences of being caught are more important, although the effect is small.

8 While other studies have used other measures, such as the percentage of correct choices, the advantage of our measure is that it is not sensitive to the frequency with which the seller has a red card. If, for instance, in a particular session 60 % of the sellers have a red card, then simply always buying a red card will mechanically lead to correct choices in 60 % of the time, while accuracy would be at chance level according to the measure we use (*D* = 0).

9 The reported nonparametric tests related to the accuracy of detecting deceit are based on taking the participant’s mean over all rounds within a treatment as the independent unit of observation.

10 In the first set of sessions we ran (in 2012), we found that the ability to detect deceit was considerably higher in the Questions treatments, with *D* = 0.18 in Q–A and *D* = 0.29 in Q–R. These findings failed to replicate in the second set of sessions (2014). If we include the sessions with participants from the experimental economics class (see footnote 5), the corresponding values for *D* are: 0.07 in NQ–A (*p* = 0.046), 0.11 in NQ–R (*p* = 0.002), 0.11 in Q–A (*p* = 0.003), 0.12 in Q–R (*p* = 0.006).

11 Of course, it is still possible that detecting deceit is easier in Q-R, but that this effect is offset by a selection effect.

12 We estimate a mixed crossed-effects model with buyer and seller random effects using the xtmixed command in Stata. The advantage of this method is that the different groups need not be nested. Estimations with (nested) session and buyer random effects yield similar results, and making the standard errors robust in those specifications does not affect the results (regression estimations are available upon request).

13 If we estimate this specification using session and buyer random effects, the coefficient of “Rich X Seller has a red card” is significant (*p* = 0.054). With robust standard errors, the coefficient is no longer significant (*p* = 0.127).

14 Since the experiment took place in a lab with open cubicles, another potential learning channel is when buyers overhear the conversation of the seller they will meet in the next round. This may be particularly relevant when sellers recommended the black product in the round before, making a recommendation to buy red in the current round less credible. We do not find any evidence of this: when we exclude sellers that recommended the black product in the round before, estimates of specification (3) in Table 3 are very similar.

15 We are grateful to an anonymous referee for pointing that out.

16 This is based on treating every action as an independent observation. Estimates from linear regressions with buyer and seller random effects yield similar conclusions.

17 Based on the binomial distribution with 9 trials (since buyers get on average 9 red recommendations) and a 0.55 success probability (the mean of 0.5 and 0.62). These calculations are admittedly a bit rough, because we do not take into account that not all buyers meet the same number of sellers recommending red, and some participants only played 9 instead of 10 rounds.
