# Supplementary material for: How private is private information? The ability to spot deception in an economic game
Source: Exp Econ. 2016 Feb 22;20(1):19–43. doi: 10.1007/s10683-015-9474-8 (PMC5326809; doi:10.1007/s10683-015-9474-8)
Supplement: Supplementary file 1 — Supplementary material 1 (DOCX 167 kb) [file 10683_2015_9474_MOESM1_ESM.docx]

**APPENDIX B: Instructions (Not for publication)**

Below are the instructions for the no-context treatment that starting with the no-questions treatment. Instructions for the sessions with the interaction treatment first were very similar with some obvious changes.

*Instructions for the no-context treatment.*

Welcome to this experiment. Please read the instructions carefully. We ask you to remain silent throughout the experiment unless indicated otherwise.

There are two types of participants in this experiment: "sellers" and "buyers." Once you have been randomly assigned a type, you will keep that same type for the entire experiment. The experiment consists of two parts, each with 10 rounds. Sellers and buyers are re-matched every round such that no two participants will ever be paired more than once with each other within each part of the experiment.

One of the rounds of the two parts will be randomly selected at the end of the experiment, and you will be paid your earnings of that round. We will not make public which round has been selected for payment, to ensure that you cannot identify your partner of that round and vice versa. You will also receive €5 as a fixed show up fee.

**Part 1.**

What follows is a description of the tasks for both types for the first 10 rounds of the experiment.

In the first round, seller 1 is matched to buyer 1, seller 2 to buyer 2, etc. The seller sits to the right of the buyer when facing the computer screen.

Sellers: sellers draw (without looking) a card from a deck of 10 cards, of which five are red and five are black. After seeing the card, the seller will give the card back, we will put it back in the deck and proceed to the next seller. The seller will keep the same color of the card for all 10 rounds. Every seller is paired with one buyer. The seller must make a recommendation to the buyer to “buy” a red card or a black card.

Buyers: The buyer does not observe which card is drawn by the seller. After the seller has made a recommendation, the buyer must decide to buy the red card or the black card.

Recommendation phase: The seller has 10 seconds to make a recommendation. The starting time and ending time are announced by the sound of a bell. Only the seller is allowed to speak during that time. Please do not speak too loudly.

During this recommendation phase, all pairs of participants stand up and face each other, as in the diagram below. The rest of the time you remain seated and silent.

buyer seller buyer seller

After the recommendation phase, sellers and buyers fill in the decision forms *in private*.

The seller writes down:

1. The number of the buyer,
2. The recommendation he or she made (black or red),
3. How certain (s)he feels that the buyer buys a black or red card (on a 10 point scale).

The buyer writes down:

1. The number of the seller,
2. The card he or she wishes to buy (black or red),
3. Which color the seller recommended,
4. How certain (s)he feels that the color of the card that the seller drew is black or red (on a 10 point scale).

Earnings: The seller earns €20 if the red card is bought by the buyer, independent of whether or not the seller actually has a red or a black card. The buyer earns €20 if he bought the card with the same color that the seller has actually drawn. Thus, the seller always earns more if the buyer buys a red card. The buyer always earns more if he buys a card of the same color as the seller drew. There are four possibilities:

1. The seller has a red card. The buyer buys the red card. Both earn €20.
2. The seller has a red card. The buyer buys the black card. Both earn nothing.
3. The seller has a black card. The buyer buys the red card. The seller earns €20, the buyer nothing.
4. The seller has a black card. The buyer buys the black card. The seller earns nothing, the buyer earns €20.

After completing the form for a round, please wait until we announce that we go to the next round. All sellers remain seated, and all buyers move to the next seller. Sellers are numbered 1 through 10. So if you are matched with seller 1 in the first round, you would then go to seller 2. After being paired with seller 10 you go to seller 1.

Here is a summary of the steps:

1. The seller draws a card,
2. When the bell rings each pair stands up facing each other, and the seller makes a recommendation during 10 seconds,
3. When the bell rings again you sit down and fill in the form in private for that round,
4. Buyers move to the next seller and we repeat steps 2 to 4.

Please always keep your sheets private so that others cannot see your decisions.

If you have any questions, raise your hand and wait for the experimenter.

**Part 2.**

What follows is a description of the tasks for both types for the next (and last) 10 rounds of the experiment.

The second part proceeds in a very similar way as the first part. The only difference is that after the recommendation phase of 10 seconds (announced by the sound of the bell), there will be another 90 seconds in which the buyer can ask questions to the seller and the seller can respond. The communication in these 90 seconds is completely free as long as no offensive, threatening, or indecent language is used. You remain standing facing your paired participant until we announce that the 90 seconds have passed at which point you sit down and complete the decision forms *in private*.

You start again in the same position as in part 1. All sellers will draw another card from the deck.

If you have any questions, raise your hand and wait for the experimenter.

*Instructions for rich-context treatment.*

The instructions for the rich-context treatment were the same except for the following:

a) Buyers were told they could buy a red or black holiday package (instead of card)

b) The part describing the task for sellers had the following additional text:

Sellers then receive a sheet with a description of two holiday packages. The two holiday packages have the same destination, but one package is more attractive for the buyer than the other. The best package for the buyer will get the label of the card drawn from the deck by the seller. Thus, if the seller draws a red card, the better value package for the buyer is identified as “package Red,” while if the seller drew a black card, the better value package for the buyer is identified as “package Black.” The seller will keep the same sheet for all 10 rounds. The holiday destination is different for each seller.

Sellers will get 5 minutes to study the sheet with the descriptions of holiday packages. They can keep this sheet with them throughout the part. An example is given at the end of these instructions.

c) The part on earnings was replaced by:

Earnings: The seller earns €20 if the red package is bought by the buyer, independent of whether or not the seller actually drew the red or the black card. The buyer earns €20 if (s)he bought the package that has the best value for the buyer. Thus, the seller always earns more if the buyer buys the red package. The buyer always earns more if he buys the best package (which is the red package if the seller drew a red card, and the black package if the seller drew a black card).

There are four possibilities:

1. The seller drew a red card (so the red package has the best value for buyer). The buyer buys the red package. Both earn €20.
2. The seller drew a red card (so the red package has the best value for buyer). The buyer buys the black package. Both earn nothing.
3. The seller drew a black card (so the black package has the best value for buyer). The buyer buys the red package. The seller earns €20, the buyer nothing.
4. The seller drew a black card (so the black package has the best value for buyer). The buyer buys the black package. The seller earns nothing, the buyer earns €20.

d) The following example of the holiday packages was provided to all participants. For every seller and every part there was a different set of holiday packages.

| **OPTION A: LOW VALUE**  **Kervansaray Lara Resort**  **Lara Beach, Turkey**  *******  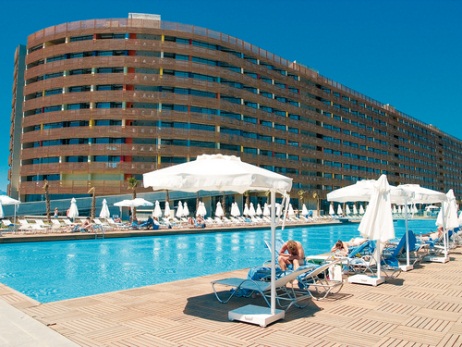  **Key Features**   - Two swimming pools - sun terraces with sunloungers and parasols - indoor pool - buffet restaurant   The Kervansaray Lara Resort is modern in style and is situated on the beachfront. This hotel has amazing architecture and good facilities and will particularly appeal to couples and families who wish to enjoy a beach holiday in a quiet resort.  **Average Customer Ratings**   - Holiday overall 71% - Accommodation 69% - Location 66% - Food 70%   **REMARKS:**  food in hotel often cold, far from the beach, no good connection to airport | **OPTION B: HIGH VALUE**  Alba Royal  Nr Side, Turkey  *****  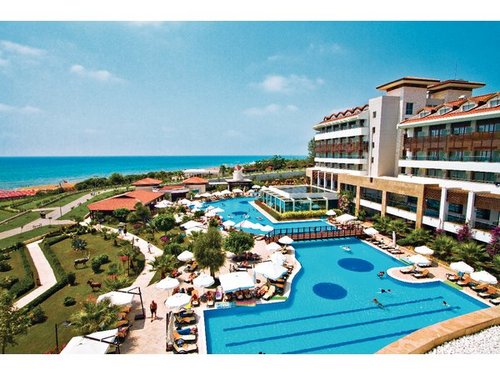  **Key Features**   - Outdoor free–form swimming pool and sun terraces with sunloungers and parasols (at least one pool open Nov–Mar) - buffet restaurant - three à la carte restaurants - 24hr room service (not part of All Inclusive)   Situated directly on the sandy beach with stunning views of the sea‚ the stylish Alba Royal offers a host of sporting activities‚ and is ideal for couples. This hotel offers bright‚ comfortable rooms and an excellent selection of facilities‚ including a choice of bars and restaurants and a modern‚ well–equipped spa.  **Average Customer Ratings**   - Holiday overall 91% - Accommodation 92% - Location 88% - Food 90% |
| --- | --- |
